# Rice price volatility and dietary diversity in Bangladeshi farm households: panel data evidence

**DOI:** 10.3389/fnut.2025.1691049

**Published:** 2026-01-12

**Authors:** Tamanna Mastura, Mohammad Jahangir Alam, Ismat Ara Begum, Md. Asif Iqbal, Andrew M. McKenzie

**Affiliations:** 1 Department of Agribusiness and Marketing, Bangladesh Agricultural University, Mymensingh, Bangladesh; 2 Department of Agricultural Economics, Bangladesh Agricultural University, Mymensingh, Bangladesh; 3 Department of Agricultural Economics and Agribusiness, University of Arkansas, Fayetteville, AR, United States

**Keywords:** rice price, dietary diversity, agricultural diversity, panel data, Bangladesh

## Abstract

**Introduction:**

Food price is an influential factor in gaining food and nutritional security. This paper estimated the association between non-seasonal rice price volatility and dietary diversity in rural Bangladesh.

**Methods:**

Using data from the Bangladesh Integrated Household Survey (2012, 2015, and 2018) combined with World Food Program price data (2012-18), we employed a fixed-effects Poisson regression model to assess how rice price volatility affects household dietary diversity.

**Results:**

The study found a statistically significant and positive correlation between rice price volatility and dietary diversity. The associations are the same for all income groups across all income quantiles; having more income doesn’t necessarily help households keep eating expensive food. Results show that a 1% increase in rice price volatility is associated with roughly a 0.8% increase in household dietary diversity, suggesting adaptive substitution behavior.

**Discussion:**

The findings suggest that farm households respond to rice price volatility by diversifying their diets rather than reducing dietary quality, likely through substitution toward alternative food groups and diversified production. Policies should balance rice intensification with agricultural diversification to support both caloric adequacy and improved nutrition.

## 1 Introduction

Food security and the volatility of food prices remain critical global challenges. Food price is one of the crucial factors of food security (1) since rapid food price variations may impact the well-being of poor households by limiting their access to adequate nutrition, resulting in reductions in food consumption, calories, protein, and dietary diversity (2). Even though households may make changes to account for seasonal food price variance, the risk and uncertainty caused by the unpredictable nature of food prices owing to unforeseen events remains a significant concern (3). Price volatility encapsulates the concept of fluctuating prices, which are increasing and decreasing. Economists commonly use this term to describe the measurement of frequent short-term fluctuations. In this context, “short-term” refers to changes in price that occur every day, week, or month (4). Since the interaction of demand and supply determines market prices, they become volatile if their relationship changes, such as when demand or supply shocks occur. The production of agricultural commodities, which serve as the basis for the production of foodstuffs, is inherently dependent on external factors such as weather and soil conditions. Food production is thus intrinsically unpredictable throughout the year, whereas consumption of staple foods is relatively stable. Apart from natural price dynamics, commodity prices could become more volatile (5). When prices move predictably due to fundamentals in the market or the changing seasons, for example, this is not necessarily a reason to be alarmed. However, price fluctuations become troublesome when they are vast and unpredictable, creating a state of unpredictability that raises risks for all actors involved in production, trade, consumption, and government, as well as the potential for sub-optimal decisions (6). Uncertainty in food consumption is a natural result of high volatility in food prices. The poor, who are already malnourished, become more vulnerable to nutritional deficiencies because of the high price volatility they face while buying food. Over the past decade, food price volatility has been a major challenge for smallholder farmers in developing countries, whose effects on smallholder well-being have been the subject of extensive discussion. There is a paradoxical connection relating food producers to food insecurity, which is highlighted by notions such as the “agriculture-nutrition disconnect” (7) and the “hungry farmer paradox” (8). Depending on household income and livelihood activities, unexpected price changes may impact food security. It has been argued that a rise in household income leads to an increase in the quantity of food consumed, and then in the quality of diet (9). If this is the case, we may assume that the diversity of the low-income people’s diets is more resilient to shocks in the price of staple foods than the high-income people’s, as they have a greater elasticity of demand for higher quality foods (10). In the event of large negative price or income shocks, there is evidence that low-income families cut back on food spending after prioritizing other necessities (11). The impact of fluctuating food prices on individual households can differ depending on whether they are net sellers or buyers of certain commodities (12–16). In developing countries, smallholder farmers are both producers and consumers of food and must buy additional foodstuffs from outside unless they are completely self-sufficient (17). Therefore, the income required to purchase food must be acquired through the selling of one’s produce in markets, wage labor, or other non-agricultural or non-farm subsistence activities (18–20). The household in the net selling position will benefit from the price increase, while the household in the net buying position will likely be dissatisfied (16). However, shifts in selling prices of food from which smallholders gain may not always reflect changes in the pricing of foods that smallholder households may need to buy (17). The issue is that households’ net selling position might change from season to season, which is also challenging to achieve, especially for low-income families who may be net sellers after harvest, as they have the option of storing food for use during the lean season or selling it at higher off-season prices to compensate for the loss of income. At the same time, they may be net buyers during the lean season when they do not have surplus produce (21). These shifts have significant consequences for food and nutrition security. Households in underdeveloped nations can operate as both net sellers and net buyers, making it far more challenging to estimate the impact of price volatility (21).

Grains are the major staple food all over the world. As a result, they are vital in the discussion of food price volatility. Rice, a type of grain, is consumed daily by most Asians (13), and the price of rice has a major impact on household food insecurity (21). Likewise, rice is the main staple food in Bangladesh, and the diet of Bangladeshi people for calorie intake depends on rice consumption. Over 70% of Bangladesh’s cropped land is dedicated to rice production, and of the country’s total dietary energy intake, 71% comes directly from rice (22). The government has enacted various rice policies to increase production and reduce reliance on foreign suppliers. Since 2004, the government has tried to maintain stable rice prices by selling the commodity on the open market (23). This was formed after food prices in Bangladesh began to rise rapidly due to the worldwide price hike. This enabled individuals across the country to buy rice at reduced prices. However, market prices have increased beyond control in the observed time frame (2016–2020), affecting both farmers and consumers (23). Because nearly all rice farmers used to sell a significant amount of marketable surplus to generate revenue. Though smallholders’ adaptive ability in the context of uncertain food price changes is critical to examine, they usually diversify their cropping patterns to be more adaptable to food price volatility if they can invest in inputs (24), which in turn offers production diversification to the farmers by providing them with new crops that they cannot obtain due to cost or poor infrastructural constraints. Moreover, rural households’ access to food relies heavily on their production, so households that combine crop and non-crop agricultural activities (for example, crop production and livestock raising) have more diverse diets (25–34). Thus, policymakers should be concerned about the effect of rice price volatility on dietary diversity, as rice is the principal staple in Bangladesh and fluctuations in its price can substantially alter household food allocation decisions. Understanding this linkage is therefore essential for designing nutrition-sensitive price-stabilization policies, targeted safety nets, and agricultural diversification programs that protect vulnerable populations from the nutritional consequences of market shock.

Considering the arguments exposed here, we assume that the non-seasonal rice price volatility may impact diet diversification because the quantity and quality of other types of food consumption may vary depending on rice price fluctuations. Although no such impact-based micro-level assessment of price volatility on household food security exists in Bangladesh, the benefit of using a rich panel dataset of 3 years from the Bangladesh Integrated Household Survey (BIHS) of the International Food Policy Research Institute (IFPRI) and the availability of authentic consumer price data from the WFP database, this study tries to find out new knowledge insights. This study is among the first to examine the association between rice price volatility and household dietary diversity in South Asia using micro-level household data. As a whole, this study attempts to assess the association between rice price volatility and dietary diversification among Bangladeshi farm households. More precisely, this study contributes by (i) focusing explicitly on rice price volatility rather than price levels, (ii) utilizing high-frequency panel data from the Bangladesh Integrated Household Survey, and (iii) linking these fluctuations directly to the Household Dietary Diversity Score (HDDS). Together, these features offer new evidence on how market instability shapes diet quality among Bangladeshi farm households.

The remainder of this paper is structured as follows: The next section, Section 2, describes the data sources and explains the empirical methods. Section 3 presents the results from the descriptive and empirical analysis, and Section 4 concludes.

## 2 Methodology

### 2.1 Data

This study combines data from the Bangladesh Integrated Household Survey (BIHS) and the World Food Programme (WFP) price database to examine the association between food price volatility and dietary diversity among farm households in Bangladesh. The BIHS, implemented by Data Analysis and Technical Assistance Limited under the supervision of the International Food Policy Research Institute (IFPRI), provides three nationally recognized waves of household-level data collected in 2012, 2015, and 2018. The survey includes 6,503 households from 325 villages across the seven administrative divisions: Barisal, Chittagong, Dhaka, Khulna, Rajshahi, Rangpur, and Sylhet.

For this analysis, households from Rangpur were excluded because consistent rice price data were unavailable in the WFP dataset for that division. The remaining six divisions represent the country’s major agro-ecological zones and key rice-producing regions, ensuring broad spatial and socioeconomic coverage. To focus on agricultural livelihoods, we retained only those households engaged in at least one production activity—crop cultivation, livestock rearing, or aquaculture.

Households that split between survey waves (2012–2018) were dropped to maintain a balanced panel consisting of the same households observed in all three waves. Although this exclusion slightly reduces representativeness, it enhances internal validity by enabling consistent within-household comparisons over time. The final analytical sample includes 3,690 farm households, yielding 11,070 individual observations per wave, with complete information on dietary diversity and socioeconomic characteristics.

While the final sample is smaller than the original BIHS, it remains broadly aligned with the survey’s rural farming population frame. Since the BIHS is designed to be nationally representative for rural areas, and the excluded households were primarily omitted due to missing price data rather than systematic bias, the results can be interpreted as representative of farm households across the six included administrative divisions of Bangladesh. Since rice is the main staple food item in the diet of the Bangladeshi people, from a food security perspective, rice is the main food source of calorie intake. According to the BIHS survey data, the respondent households reported that rice was the most common food item in the 7-day recall period, which is a proxy for a regular dietary pattern, and households consume coarse rice. Thus, this study has focused on the calculation of the price volatility of domestic rice, and the historical price data of coarse rice from the years 2011 to 2018 were collected from a secondary source. The World Food Program (WFP) collects the average retail market price of coarse rice from the six divisions (Barisal, Chittagong, Dhaka, Khulna, Rajshahi, and Sylhet) of Bangladesh, and these data are available on the WFP’s website. To ensure the availability of data from all years and divisions, this study used the WFP rice price data to calculate the price volatility of domestic coarse rice over 8 years. Since we do not have more extensive information, the method includes these divisional prices as a stand-in for local prices. The BIHS surveys were conducted from October to March and did not collect consumption data with seasonal variation. Verbal informed consent was obtained from all participants before the interview. The BIHS surveys had to rely on the memory of the respondents.

Although division-level price data may not fully capture the heterogeneity of price shocks experienced by individual households within each division, it represents the most consistent and reliable time-series source available for Bangladesh during the study period. We acknowledge, however, that this aggregation may obscure localized market fluctuations and introduce measurement error into the volatility variable. This error is expected to be classical in nature, likely biasing the estimated association toward zero, so our findings should be interpreted as conservative estimates of the true relationship between rice price volatility and household dietary diversity.

### 2.2 Measuring dietary diversity

We utilize the dietary recall data (previous 7-day recall period) to determine how frequently different families consume different types of food, as it is a good method for capturing how households could respond to fluctuating food prices (35, 36). In the face of ever-increasing prices for staple foods, many families have chosen to consume a variety of foods (37, 38). According to FAO (39), dietary diversity is a qualitative indicator of food consumption and serves as a proxy for nutrient adequacy. The Household Dietary Diversity Score (HDDS) is widely applied for this purpose and has been validated as a reliable indicator of both per capita energy availability and household economic access to food (29). Empirical evidence shows that dietary diversity is positively associated with income and micronutrient sufficiency, supporting its use as an indicator of food security (36, 40, 41). While the strength of the correlation between dietary diversity scores and nutrient adequacy differs by country and context, studies have demonstrated that measures such as the household dietary diversity score are effective tools for assessing food insecurity across regions (36, 42, 43). Unlike simply counting the total number of foods consumed, the HDDS records the consumption of distinct food groups by any household member during the reference period. According to FAO (39), there are twelve food groups used to calculate the HDDS: cereals, roots and tubers, vegetables, leafy vegetables, fruits, meat and poultry, eggs, fish and seafood, pulses/nuts, milk and milk products, oil/fats, sugar and honey, which used to calculate the HDDS, and it varies from 0 to 12; 12 means maximum diversity and 0 means no diversity. These food groups were used in the BIHS module to collect dietary information for each household. Each food group counts toward the household score if a food item from the group was consumed by anyone in the household in the previous 7-day recall period. Using the HDDS, we can also categorize household’s access to food as follows: those who consume eight or more food groups are considered to be food secure, those who consume four to eight food groups are considered to be moderately food secure, and those who consume less than four food groups are considered to be food insecure (44–46).

### 2.3 Measuring rice price volatility

Volatility estimates are based on the presumption that the real, deflated price of food, P, exhibits three distinct components: a trend component, *τ*, a seasonal component, *η*, and an unexplained error component, *ε*. That is, *P* = f (τ, η, ε) (21). Non-seasonal price volatility, denoted by the unexplained factor, ε, is under consideration in this study.

Since this research attempt is dedicated to elucidating the impact of price volatility, the deflated data have been detrended to verify that the unexpected price volatility has been properly identified. This study started by detrending the historical rice price record (21). So, creating a continuous time variable is the initial step, mentioned as, $t_{m,y}$ , and considering time = 1 for Jan 2011, = 2 for Feb 2011, … = 96 for Dec 2018 and then regress the deflated prices, $P_{d,m,y}^{x}$ , for commodity x (rice), in month m of year y, and division d, on the time variable, $t_{m,y}$ . In equation form (Equation 1):


$$P_{d,m,y}^{x}=\alpha_{d}^{x}+\beta_{d}^{x}t_{m,y}$$ (1)

The detrended deflated prices, $R_{d,m,y}^{x}$ , are then obtained by subtracting the fitted price values from the observed prices for commodity x (rice), generating the linear forecast of the deflated prices, $\;\hat{P_{d,m,y}^{x}\;}$ (Equation 2), as follows (21):


$$R_{d,m,y}^{x}=P_{d,m,y}^{x}-\hat{P_{d,m,y}^{x}}$$ (2)

Then, after centering the prices to obtain the price deviation, $\mathrm{de}v_{d,m,y}^{x}$ , we divided the remaining food price variation into its seasonal and volatility components, respectively (Equation 3), as follows (21):


$$\mathrm{de}v_{d,m,y}^{x}=R_{d,m,y}^{x}-{\bar{R}}_{d}^{x}$$ (3)

Where $R_{d,m,y}^{x}$ is the deflated, detrended price and ${\bar{R}}_{d}^{x}$ is the overall average price for commodity x (rice) in division d, across all months and years. Then add and subtract the average monthly price of commodity x (rice) in division d, ${\bar{R}}_{d,m}^{x}$ , from the price deviation, $\mathrm{de}v_{d,m,y}^{x}$ , as follows:


$$\mathrm{de}v_{d,m,y}^{x}=R_{d,m,y}^{x}-{\bar{R}}_{d}^{x}+{\bar{R}}_{d,m}^{x}-{\bar{R}}_{d,m}^{x}$$ (4)

Rearranging Equation 4 gives:


$$\mathrm{de}v_{d,m,y}^{x}={\bar{R}}_{d,m}^{x}-{\bar{R}}_{d}^{x}+R_{d,m,y}^{x}-{\bar{R}}_{d,m}^{x}$$ (5)

In Equation 5, the first two variables define the seasonal price variation, $\eta_{d,m}^{x}$ = ${\bar{\;R}}_{d,m}^{x}-{\bar{R}}_{d}^{x}$ , and the last two variables define the price volatility component of interest, $\varepsilon_{d,m,y}^{x}\;$ = $\;R_{d,m,y}^{x}-{\bar{R}}_{d,m}^{x}$ (21). After simplifying as Equation 6:


$$\mathrm{de}v_{d,m,y}^{x}=\eta_{d,m}^{x}+\varepsilon_{d,m,y}^{x}$$ (6)

Finally, we used the household interview month with year and census division to match the decomposed price series to the household data.

### 2.4 Model specification

To investigate the association between food price volatility and household dietary diversity, this study employed a Poisson regression model, as the dependent variable (HDDS) is a count variable, while household-level fixed effects account for time-invariant unobserved heterogeneity. Since the data in this study are panel data, a household fixed effects model was employed to account for time-invariant household-level heterogeneity. Year dummies were also included to capture time-varying macro shocks, such as climatic events or policy changes, thereby minimizing omitted-variable bias. Moreover, the fixed effects model is essential for isolating the true effect of rice price volatility on dietary diversity by removing bias arising from unobserved, time-invariant household characteristics. The model was constructed in the following way:


$$Y_{i,d,y}=\beta_{0}+\beta_{1}\varepsilon_{d,m,y}+\beta_{2}M_{i,d,y}+\beta_{3}X_{i,d,y}+u_{i,d,y}$$

Where, *Y*_*i*,*d*,*y*_ = Household dietary diversity score (HDDS) in household *i* in different divisions and years.

*Ɛ*_*d*,*m*,*y*_ = Price volatility component of rice in different divisions and years.

*M*_*i*,*d*,*y*_ = Vector of farm characteristics and market access indicator of household i in different divisions and years, such as production diversification score, farm income, distance to the closest market, market participation and non-farm income.

*X*_*i*,*d*,*y*_ = Vector of other household characteristics, such as the sex of household head, age of household head, education of household head, earning status of women, education of women, household size, dependency ratio, etc.

*μ*_*i*,*d*,*y*_ = error term.

It’s not easy to draw a straight line between household dietary diversity status and food price effects. However, the time-varying unobservable elements, such as other macroeconomic events to which households may respond differently in terms of their dietary diversity status and food price volatility, are not taken into account by the fixed effects model (47). In particular, we focus on the coefficient of interest, β1, which represents the connection between dietary diversity and non-seasonal food price volatility for households.

## 3 Results and discussion

### 3.1 Descriptive statistics and sample characteristics

Before doing an econometric analysis, a descriptive analysis is conducted to understand the nature and pattern of the data. To characterize descriptive statistics of three waves of the panel, this study has also used parametric tests to compare the means of related variables.

Table 1 results show the changes and average value of various socio-economic characteristics of the sampled farm household over time, implying that differences over time are significant. The production diversification score, which is the total number of different food crops and animal products produced by each household, is used as an explanatory variable to represent the extent of farm diversification (28, 46, 48–51). The analysis of production diversification scores throughout the three observed periods reveals a dynamic trend characterized by fluctuations. Notably, scores rose significantly from 2012 to 2015, followed by a subsequent decline by 2018. This observation implies a constantly changing agricultural scenario in terms of the variety of crops cultivated by rural households in Bangladesh. The observed mean differences suggest that there have been substantial changes in production diversification over time, and these changes are statistically significant.

**Table 1:** Descriptive statistics of explanatory variables.

Explanatory variables	Mean and standard deviation (SD)				Mean diff. (2012 vs. 2015)	Mean diff. (2015 vs. 2018)	Mean diff. (2012 vs. 2018)
	Pooled	Wave 1	Wave 2	Wave 3			
		2012	2015	2018			
Production diversification score	8.039 (5.232)	7.996 (5.669)	7.533 (4.828)	8.587 (5.113)	−0.463*** (0.121)	1.054*** (0.121)	0.591*** (0.121)
Sex of HH head (dummy)	0.806 (0.395)	0.820 (0.384)	0.807 (0.394)	0.791 (0.407)	−0.013 (0.009)	−0.016 (0.009)	−0.029*** (0.009)
Education of HH head (years)	3.632 (4.115)	3.510 (4.091)	3.630 (4.082)	3.756 (4.169)	0.119 (0.096)	0.126 (0.096)	0.246** (0.096)
Earning Status of women (dummy)	0.756 (0.430)	0.637 (0.481)	0.761 (0.427)	0.870 (0.336)	0.124*** (0.010)	0.109*** (0.010)	0.233*** (0.010)
Education of women (years)	3.402 (3.607)	3.234 (3.564)	3.374 (3.569)	3.599 (3.680)	0.140 (0.084)	0.225** (0.084)	0.366*** (0.084)
Household size (number)	4.817 (1.852)	4.209 (1.528)	4.819 (1.749)	5.423 (2.042)	0.609*** (0.042)	0.604*** (0.042)	1.214*** (0.042)
Child share in HH size (%)	35.003 (21.014)	39.129 (21.675)	35.503 (20.589)	30.376 (19.813)	−3.626*** (0.482)	−5.128*** (0.482)	−8.753*** (0.482)
Elder member share in HH size (%)	14.855 (20.411)	6.325 (15.188)	14.234 (19.035)	24.007 (22.362)	7.909*** (0.444)	9.773*** (0.444)	17.682*** (0.444)
Market distance (Km.)	1.756 (2.708)	1.702 (1.679)	1.690 (1.849)	1.875 (3.967)	−0.012 (0.063)	0.185* (0.063)	0.172* (0.063)
Market participation (dummy)	0.592 (0.491)	0.637 (0.481)	0.574 (0.495)	0.564 (0.496)	−0.063*** (0.011)	−0.010 (0.011)	−0.073*** (0.011)
Annual Farm income (Tk.)	35137.840 (67668.32)	27408.650 (50720.55)	34109.940 (65631.52)	43894.920 (81987.17)	6701.292*** (1567.624)	9784.982*** (1567.624)	16486.270*** (1567.624)
Annual non-farm income (Tk.)	63018.870 (99146.89)	41136.960 (59758.08)	62594.970 (105989.0)	85324.680 (117107.40)	21458.01*** (2269.902)	22729.71*** (2269.902)	44187.72*** (2269.902)
HDDS	10.266 (1.500)	9.751 (1.574)	10.448 (1.401)	10.600 (1.378)	0.697*** (0.034)	0.151*** (0.034)	0.849*** (0.034)
Rice price volatility	−30.410 (1.644)	−31.311 (0.973)	−30.350 (0.904)	−29.568 (2.195)	0.961*** (0.034)	1.743*** (0.034)	0.783*** (0.034)
Number of observations (HH group)	11,070	3,690	3,690	3,690	–	–	–

*t*-values are in parentheses. ***p < 0.01, **p < 0.05, *p < 0.1.

It was discovered that nearly 40 % of the sample farm households were in a net selling position due to the fact that they relied on their production for rice consumption, whereas the remaining farm households were reported as net buyers due to the fact that they purchased rice from the market for their household consumption. The market distance exhibits a marginal upward trend between the years 2012 and 2018. The observed mean differences indicate that, as time progresses, households are encountering somewhat increased distances to access markets. This trend may potentially impact the overall accessibility and pricing of items. The study period reveals a noticeable decline in market participation, as indicated by the presence of negative mean differences. This observation implies a possible change in the level of involvement of agricultural households in commercial transactions, which could have an impact on their financial earnings as well as their dietary preferences. Though the market participation rate decreases over time, farm income increases significantly each year. The observed mean differences in yearly farm income demonstrate a notable and positive trend of growth during the period from 2012 to 2018. On the other hand, the income from non-farm sources also increases significantly. The positive mean differences indicate a significant rise in yearly non-farm income. This implies that households have expanded their sources of income outside agriculture, enhancing their overall economic resilience. Considering the household characteristics, around 80 percent of these households’ heads are found to be male members. The positive mean differences in the education level of the household head suggest a consistent improvement in the educational attainment of household heads, potentially impacting decision-making processes and resource management within the households. However, the earning status of women exhibits a favorable trend, characterized by a notable rise from 2012 to 2018. This observation indicates a notable trend in which an increasing number of women residing in agricultural households are making significant economic contributions.

### 3.2 The association between rice price volatility and household dietary diversity

The HDDS is a metric used to assess the variety and quality of food consumed within a household. The observed rise in the score over successive waves of data collection suggests a positive development in the range and diversity of food items consumed by households. The presence of positive mean differences highlights the existence of a positive pattern in dietary variety, which may be impacted by a range of socioeconomic factors and market conditions. On the other hand, the observed negative mean differences in rice price volatility indicate a progressive volatility decline throughout the study. The decrease in volatility has the potential to provide economic stability for agricultural households, thereby influencing their capacity to strategize and allocate resources.

### 3.3 Heterogeneity of effects: the role of income

This study assumed that the impact of rice price volatility on household dietary diversity status may also vary by household income level. To check this assumption, the farm-households were divided into five groups according to their annual income quantiles (Table 2), where each group represents the information of 20% of households depending on the income range. The inclusion of income quantiles is crucial in examining the varying effects of rice price volatility on dietary diversity among different income groups. This approach allows for a more comprehensive comprehension of the economic determinants that shape eating patterns within farm households.

**Table 2:** Income quantile group.

Quantile group	Quantile range (TK.)	% of median	Share, %	L(p), %	GL(p)
Q1 (Bottom 20%)	<31,800	33.830	1.644	1.644	2223.591
Q2	31,800–72,000	76.596	7.859	9.503	12852.302
Q3	72,000–120,000	127.660	14.287	23.790	32173.923
Q4	120,000–200,400	213.191	22.536	46.326	62651.320
Q5 (Top 20%)	>200,400		53.674	100.000	135240.010

Share = quantile group share of total annual income; L(p) = cumulative group share; GL(p) = L(p)*mean (annual income).

The first quartile, representing the bottom 20% income group, exhibits the lowest percentage of total income at 1.644% and provides the smallest value to the cumulative income, denoted as GL(p) = 2223.591. The top quintile (Q5) of income earners possesses the largest proportion of total income, accounting for 100% of the overall income distribution. Additionally, this income group makes the highest cumulative contribution to the total income, amounting to GL(p) = 135,240.010. The income groups ranging from the second to the fourth quartile exhibit a gradual rise in their proportion of total income and cumulative income contributions. This trend signifies a progressive distribution of income among quantile groups.

**Figure 1:**
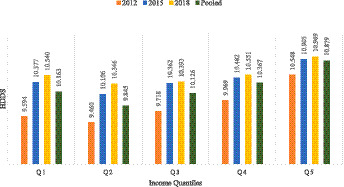
HDDS according to income quantiles.

The main outcome variable of this study is the household dietary diversity score. Following the income quantiles, the HDDS of the sampled farm households was assessed, which is shown in Figure 1.

The HDDS of different farm-household groups based on the income quantiles differ among groups and, in general, increase over the years. The differences are found to be statistically significant. Usually, the top 20% of households, which are in the higher income group, have the overall highest HDDS, but the lowest HDDS has been found among the households in the second quantile (Q2), which are in the second lowest income group. The bottom 20% of households, the lowest income group, have higher HDDS than Q2 in each wave. There are significant changes in dietary diversity among all the quantiles, and the highest difference (0.716) has been found between the top and bottom-income households. In the case of different waves, the highest significant difference (0.946) was found within households in the first quantile (Q1) from 2012 to 2018.

However, the key objective of this study is to investigate the effect of unexpected price volatility on the dietary diversity outcome. The analysis of price movements may focus only on the main food staples as they represent an important portion of the dietary requirements in developing countries. Thus, this paper has depicted the results of testing the hypothesis of whether unexpected price volatility of the main staple food may influence households to consume other food groups.

**Figure 2:**
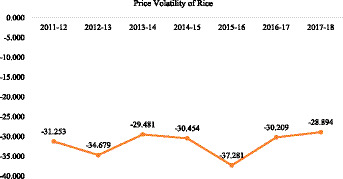
Changes in average rice price volatility component from 2011 to 2018.

For a specific commodity to be considered highly volatile, its price must experience large swings between highs and lows over a relatively short time frame. A commodity is said to have low volatility if its price increases or decreases slowly or maintains a consistent level. However, the main explanatory variable of our stated model is the rice price volatility component, which is the unanticipated price volatility component and is obtained by detrending the deflated data. The rice price volatility in Bangladesh from 2011 to 2018 is shown in Figure 2. The values range from −31.253 to −28.894, indicating the degree of volatility in the price of non-seasonal rice for each specific year. Whether the value is positive or negative, a larger absolute value indicates higher levels of volatility. Over the study period, negative values represent deviations below the long-term mean of detrended prices, rather than negative volatility. Smaller absolute values indicate lower price volatility. More specifically, from 2011–12 to 2017–18, there was a declining trend in the non-seasonal volatility component. The negative values signify that when seasonal fluctuations are accounted for, the observed rice prices for each year were, on average, lower than the aggregate average price for all months and years. This implies a level of consistency or a decrease in unforeseen fluctuations in prices when examining particular years in comparison to the mean. With the possible exception of a rise in 2015–16, the consistent negative values over the years indicate that rice price non-seasonal volatility decreased during the study period. This trend may reflect better market stability or factors influencing the lessening of unforeseen fluctuations in rice prices.

The potential positive consequences for both producers and consumers could arise from a decline in the volatility of rice prices. Producers tend to gain advantages from a more consistent and predictable income, whereas consumers may encounter reduced levels of uncertainty regarding the pricing of this staple food item. A higher price of rice always encourages rice farmers to sell their produce in the market, hoping to get higher profits from this trade. The effect of increasing the price of rice generally influenced the rice farmers to sell their marketable surplus produces in the market as they will get higher prices or profit from the trade. That cash or profit can increase their purchasing power for fulfilling their other needs, which may increase their capability to purchase more nutrient-oriented, diversified food items from the market and thus diversify their diet. As a result, getting higher profits from these kinds of trade increases their purchasing power and enables them to buy more nutrient-oriented food items from the market, thus helping them diversify their diet. On the other hand, the potential benefits of lower-than-average pricing for consumers include increased affordability of staple foods. Nevertheless, persistent declines could also indicate economic challenges for rice producers and traders.

### 3.4 Regression outcomes and effects of other determinants

The results in Table 3 show a significant positive association between rice price volatility and household dietary diversity. The coefficient for rice price volatility is 0.008 and statistically significant at the 1% level, suggesting that, on average, an increase in rice price volatility is associated with a positive change in household dietary diversity. This relationship remains robust even after accounting for household fixed effects. These results are consistent with Amolegbe et al. (21), who observed that when domestic rice price volatility rises (or falls), cereal intake declines (or increases), while consumption of other food groups rises (or falls), thereby improving or compromising overall dietary diversity. This seemingly paradoxical finding indicates that households tend to diversify their diets in response to increased rice price volatility (21, 52). Understanding this behavior is crucial for comprehending the adaptive strategies that Bangladeshi agricultural households employ to cope with economic uncertainty linked to rice prices. One potential explanation for this pattern is that increased volatility in essential food commodities, such as rice, motivates households to seek alternative food sources, thereby increasing dietary diversity. Economic theory suggests that households experiencing uncertainty often attempt to reduce risks by diversifying consumption and purchasing patterns (53), which can be observed in their dietary choices.

**Table 3:** Effect of rice price volatility on HDDS (fixed-effect Poisson model).

Variables	HDDS	
	Pooled	Fixed-effect
Rice price volatility	0.008*** (0.001)	0.008*** (0.001)
Production diversification score	0.002*** (0.00026)	0.002*** (0.00037)
Market distance	−0.001 (0.001)	−0.001* (0.001)
Market participation (1 = Yes)	0.002 (0.003)	0.006 (0.004)
Farm income	1.27e-07*** (0.000000017)	7.13e-08*** (0.000000023)
Non-farm income	8.94e-08*** (0.000000014)	7.02e-08*** (0.000000017)
HH head sex (1 = Male)	−0.019*** (0.004)	−0.004 (0.007)
HH head education	0.004*** (0.00038)	0.001 (0.001)
Earning status of women (1 = Yes)	0.018*** (0.003)	0.025*** (0.004)
Women education	0.005*** (0.0032)	0.002** (0.001)
HH size	0.013*** (0.001)	0.024*** (0.002)
Share of children	−4.320e-04*** (0.000081)	−0.001*** (0.00013)
Share of elders	−2.480e-04*** (0.000095)	−1.458e-04 (0.00014)
Constant	2.482*** (0.028)	–
Observations	11,070	11,070
Number of HH	–	3,690

Robust standard errors are in the parentheses; *** *p* < 0.01, ** *p* < 0.05, * *p* < 0.1.

The coefficient of production diversification is positive (0.002) and statistically significant at the 1% level in both models, indicating that greater diversification in farm production is associated with higher household dietary diversity. This finding aligns with Molitor et al. (24), who suggest that price volatility may encourage households to diversify both production and consumption. It can thus be inferred that unexpected short-term price fluctuations in staple foods may motivate households to increase both production and dietary diversification. The fixed-effect model reveals that market distance has a negative and statistically significant effect on HDDS (−0.001, significant at 5%), implying that households located farther from markets tend to have lower dietary diversity. In contrast, the coefficient for market participation is positive (0.002) but statistically insignificant in the pooled model, indicating that market participation is not significantly associated with changes in HDDS. However, under the fixed-effect specification, the coefficient increases to 0.006 and is statistically significant at the 1% level. This suggests that, within households over time, greater market participation is associated with higher dietary diversity. These results support the argument that market engagement provides households with access to a wider variety of foods and additional income opportunities. Islam et al. (54) similarly found that households participating in product markets tend to have more diverse diets, although increased market distance can negatively affect dietary diversity.

The coefficients for both farm and non-farm income are positive and statistically significant in both models, indicating that increases in either source of income are associated with higher dietary diversity. This finding is consistent with Okeke-Agulu & Ojeifo (55), who reported that higher annual farm and non-farm incomes significantly shift household diets toward more diversified food groups. Notably, non-farm income appears particularly important in buffering households against agricultural shocks and enhancing their ability to purchase food, thereby contributing to improved dietary diversity.

Additionally, the coefficients for household demographic characteristics, including the sex of the household head, the educational level of the head, women’s earning status, and women’s education, show significant associations with changes in HDDS in both models. For instance, the negative coefficient for the male-headed household indicates that, on average, male-headed households tend to have lower dietary diversity. Household composition also plays an important role. Household size is positively associated with dietary diversity, indicating that larger households tend to consume a wider variety of foods. The proportion of children and elderly members in a household is correlated with variations in HDDS. For example, a positive coefficient for household size indicates that, on average, larger households tend to have more diverse diets. Female-headed households are found to exhibit higher dietary diversity, and increases in women’s education amplify their influence on diet diversification, particularly when rice prices are higher. While a greater number of children or elderly members generally reduces dietary diversity, this effect is less pronounced under the fixed-effects specification. Overall, larger households or those with more adult members tend to achieve greater diet diversification, consistent with the findings of Okeke-Agulu & Ojeifo (55).

In brief, the findings of this study indicate that multiple factors, including rice price volatility, production diversification, market distance, market participation, and various socioeconomic characteristics, significantly influence the Household Dietary Diversity Score. The inclusion of household fixed effects strengthens the analysis by controlling for unobserved, time-invariant household-level factors, thereby enhancing the robustness of the observed relationships.

The findings from the fixed-effect Poisson regression analysis in Table 4 shed light on the varying impact of rice price volatility on the Household Dietary Diversity Score (HDDS) across different income quantiles (Q1 to Q5), hence offering valuable insights into the link between these variables across households with different income levels. The coefficient of rice price volatility exhibits a constant upward trend and holds statistical significance across all income quantiles, ranging from Q1 to Q5. This observation suggests a strong and reliable positive association between rice price volatility and the HDDS. The coefficients for rice price volatility across different income quantiles (Q1 to Q5) demonstrate a consistent positive relationship with dietary diversity. Although the coefficients show a high degree of similarity in magnitude across income quantiles, a nuanced distinction can be observed, as the coefficient for Q2 appears to be slightly lower in comparison to the coefficients for the remaining quantiles. Households in the second income quantile may experience income instability—slightly above the poorest but without sufficient reserves—making them less responsive to price fluctuations compared with both the poorest (Q1) and richer groups (Q3–Q5). This finding suggests that the association between volatility in rice prices and the diversity of diets is somewhat less evident for households in the second income quantile when compared to other income groups. The coefficient exhibits a magnitude of roughly 0.008 across all income quantiles. This suggests a positive correlation exists between an increase in rice price volatility and a corresponding increase of around 0.8% in the HDDS for households across all income quantiles.

**Table 4:** Effect of rice price volatility on HDDS across different income quantiles (fixed-effect Poisson model).

Variables	HDDS				
	Q1	Q2	Q3	Q4	Q5
Rice price volatility	0.008*** (0.001)	0.007*** (0.001)	0.008*** (0.001)	0.008*** (0.001)	0.008*** (0.001)
Production diversification score	0.002*** (3.749e-04)	0.002*** (3.735e-04)	0.002*** (3.750e-04)	0.002*** (3.742e-04)	0.002*** (3.747e-04)
Market distance	−0.001* (0.001)	−0.001* (0.001)	−0.001* (0.001)	−0.001* (0.001)	−0.001* (0.001)
Market participation (1 = Yes)	0.006 (0.004)	0.006 (0.004)	0.006 (0.004)	0.005 (0.004)	0.006 (0.004)
Farm income	6.99e-08*** (2.32e-08)	5.44e-08** (2.29e-08)	7.23e-08*** (2.31e-08)	7.07e-08*** (2.30e-08)	4.05e-08* (2.42e-08)
Non-farm income	6.92e-08*** (1.73e-08)	5.72e-08*** (1.58e-08)	7.11e-08*** (1.74e-08)	7.06e-08*** (1.72e-08)	4.47e-08*** (1.61e-08)
HH head sex (1 = Male)	−0.005 (0.007)	−0.002 (0.007)	−0.005 (0.007)	−0.006 (0.007)	−0.004 (0.007)
HH head education	0.001 (0.001)	0.001 (0.001)	0.001 (0.001)	0.001 (0.001)	0.001 (0.001)
Earning status of women (1 = Yes)	0.025*** (0.004)	0.024*** (0.004)	0.025*** (0.004)	0.025*** (0.004)	0.025*** (0.004)
Women education	0.002** (0.001)	0.002* (0.001)	0.002** (0.001)	0.002** (0.001)	0.002** (0.001)
HH size	0.023*** (0.002)	0.023*** (0.002)	0.024*** (0.002)	0.023*** (0.002)	0.023*** (0.002)
Share of children	−0.001*** (1.349e-04)	−0.001*** (1.347e-04)	−0.001*** (1.348e-04)	−0.001*** (1.348e-04)	−0.001*** (1.346e-04)
Share of elders	−1.435e-04 (1.434e-04)	−1.676e-04 (1.431e-04)	−1.444e-04 (1.431e-04)	−1.377e-04 (1.428e-04)	−1.366e-04 (1.431e-04)
Income quantile (dummy)	−0.002 (0.005)	−0.022*** (0.004)	0.003 (0.004)	0.011*** (0.004)	0.015*** (0.004)
Observations	11,070	11,070	11,070	11,070	11,070
Number of HH	3,690	3,690	3,690	3,690	3,690

Robust standard errors are in the parentheses; *** *p* < 0.01, ** *p* < 0.05, * *p* < 0.1.

The findings of this study reveal that there is a positive and statistically significant relationship between the production diversification score and the HDDS across all income quantiles. Specifically, the results indicate that a one-unit increase in production diversification is consistently related to a positive change in HDDS. The statistical significance of the negative coefficient for market distance is observed at the 0.1 level across all income quantiles, except for Q2, where it does not reach statistical significance. This finding implies that, on average, an expansion in market distance is correlated with a reduction in household daily dietary energy supply (HDDS) for the majority of income groups. The coefficient of market participation exhibits a positive value, while its statistical significance demonstrates variability across different income quantiles. Market participation is found to be statistically insignificant for Q1, Q2, and Q3. However, for Q4 and Q5, market participation is observed to be statistically significant at the 0.1 and 0.05 levels, respectively. The correlations for both farm and non-farm income exhibit positive and statistically significant values across all income quantiles, suggesting that increased levels of both forms of income are linked to greater dietary diversity. The coefficients of demographic factors, such as the gender of the head of household, educational attainment, the earning status of women, women’s educational level, household size, the proportion of children, and the proportion of elderly individuals, exhibit variations between income quantiles. However, these coefficients consistently demonstrate observable patterns. In numerous instances, the coefficients show statistical significance, suggesting these variables’ influence on households’ dietary diversity. The coefficients associated with income quantile dummies offer valuable insights into the impact of belonging to a particular income quantile on HDDS, relative to the reference group (Q1). As an illustration, the presence of a negative coefficient for Q2 implies that individuals belonging to the second income quantile exhibit a reduction in HDDS relative to Q1. On the other hand, positive coefficients for the higher income quantiles (Q4 and Q5) indicate an increase in HDDS compared to Q1.

Collectively, the effect of price volatility on household dietary diversity across different income quantiles shows a positive and significant association. Households that have diversified farm production, market participation, and working and educated women have found with significant association of these indicators with household dietary diversification. Households having non-farm income sources also support diversifying their diets under unexpected price volatility conditions. So, results indicate that improving income condition, or in other words, having more income, does not necessarily support the households to continue the consumption of costly food items even if it is the main food, and instead creates the tendency for having more diversified food to minimize the impact of price changes.

## 4 Conclusion

Agriculture is central to low-income countries, creating a policy tension between producer benefits from high prices and consumer gains from low prices. Food security is further threatened by price volatility, supply disruptions, and climatic shocks (56), while the relationships among import dependence, poverty, and food crises remain complex (57). Dietary diversity as a strategy can successfully ensure food security and improve nutritional security. Thus, we attempted to examine the dependency of dietary diversity on the price volatility of food components. According to our study, rice price volatility positively and significantly affects household dietary diversity. The association is the same for all income groups across the different income quantiles, meaning that improving income condition or in other words, having more income, does not necessarily support the households to continue the consumption of costly food items even if it is the main food, and instead creates the tendency for having other diversified food groups to minimize the impact of price changes. Increasingly, this is due in large part to the fact that farm households are diversifying their production. We found that the diets of farming households were more diversified as a result of increased production diversification. As time goes on, we see a steady fall in the percentage of farm households that actively participate in the market. As the price of rice fluctuates, farms are looking for ways to diversify their production by producing other high-value crops to avoid the risk, which boosts their farm income while discouraging them from relying solely on the market for their food sources and instead pushing them to consume more of what they have grown themselves. Women’s education and economic participation also contribute to expanding household dietary diversity.

This research is a valuable contribution to the existing knowledge on the influence of economic factors, specifically fluctuations in rice prices, on the dietary diversity of agricultural households in Bangladesh. The identification of a positive correlation motivates more investigation into the underlying behavioral and economic factors that contribute to these dynamics. This lays the foundation for the development of specific interventions aimed at improving food security and nutrition in the region.

### 4.1 Policy implications

The findings of this study have important policy implications for promoting food security and nutrition in rural Bangladesh. Policymakers should prioritize stabilizing rice prices, as volatility directly affects household nutrition, through measures that enhance market efficiency to reduce short-term price fluctuations. Efforts to improve market accessibility and infrastructure can further enable households to procure a wide variety of foods, mitigating the negative effects of greater market distance. Policies should also promote balanced agricultural diversification alongside rice intensification to meet caloric and nutritional needs while increasing farmers’ incomes. Supporting women’s education and income participation can enhance dietary diversity and strengthen overall food security outcomes. These results suggest that policies aiming at improving dietary diversity should not focus solely on rice self-sufficiency but should also encourage diversified agricultural production and explicitly empower women through education, access to resources, and income-generating opportunities. Overall, empowering women farmers and female household members emerges as a critical pathway for nutrition-sensitive agricultural policies to improve household diets in Bangladesh. Such interventions are closely tied to broader development goals, highlighting the importance of strategies that balance rice intensification with agricultural diversification to achieve both nutritional adequacy and income growth.

While the findings of this study provide valuable insights, it is important to acknowledge that contextual factors not included in the analysis may also influence dietary diversity. Although the use of a balanced panel enhances internal validity, the results are expected to be robust in an unbalanced sample; future studies could explicitly test this assumption. Further research is needed to gain a more comprehensive understanding of households` actual food choices in response to rice price volatility, as well as the potential influence of cultural and geographical factors on dietary habits. Additionally, integrating econometric approaches with decision-support frameworks, such as resilience modeling or stochastic optimization, could help simulate household responses under dynamic price scenarios and inform more effective policy design.

## Data Availability

Publicly available datasets were analyzed in this study. This data can be found at: https://doi.org/10.7910/DVN/NXKLZJ.
